# Disability, Genetic Counseling, and Medical Education: From Eugenics to Anti-Ableism

**DOI:** 10.1146/annurev-genom-022024-010951

**Published:** 2025-03-27

**Authors:** Cassie Houtz, Rebecca Mueller

**Affiliations:** 1 Department of Medical Ethics and Health Policy, Perelman School of Medicine, University of Pennsylvania, Philadelphia, Pennsylvania, USA; 2 Master of Science Genetic Counseling Program, Perelman School of Medicine, University of Pennsylvania, Philadelphia, Pennsylvania, USA

**Keywords:** ableism, communication, disability, ethics, genetic counseling, medical education

## Abstract

Genetic counselors have a complex relationship with disability communities due to both the legacy of eugenics and their ongoing role counseling families about prenatal testing. Drawing on a social model of disability and highlighting mistaken assumptions about quality of life for people with disabilities, scholars and activists have raised concerns about genetic technologies that strive to eliminate disability. We review the disability rights critique of prenatal screening and emphasize its ongoing relevance to genetic counseling. We then consider disability perspectives on prognostication in genetics and highlight disability-informed critiques of gene therapies. We close by reviewing efforts by, and opportunities within, the genetic counseling profession to center the perspectives of people with disabilities in genetic counseling practice and education.

## BACKGROUND

In recent years, bias against people with disabilities in medical research and practice has gained greater visibility as a source of health disparities and discrimination. Several federal organizations have issued statements and policies to begin to address this problem. In 2023, the National Institutes of Health (NIH) designated people with disabilities as a population with health disparities, recognizing the need to understand and address barriers to healthcare faced by people with disabilities (88). Additionally, the NIH proposed a revision of its mission statement that removes the phrase “reducing disability” (59, 66), which could imply that disabled people are flawed and need to be “fixed” (144). In 2024, the US Department of Health and Human Services finalized a rule to strengthen protections against disability discrimination that specifically addresses discrimination and accessibility barriers in medicine (145). Organizations focused on genetics have also begun to articulate commitments to be more intentional and inclusive with respect to disability (17, 87, 108, 147). However, we are finalizing this article in early 2025, and we are aware that the official position of the NIH may change under the leadership of the Trump administration. Nonetheless, we strongly affirm the pressing need for increased access to unbiased healthcare for people with disabilities.

Disability is a heterogeneous category and notoriously difficult to define (128). Within medicine, it typically refers to physical or cognitive impairments that make it more challenging for a person to perform certain activities and “interact with the world around them” (27). To legally qualify for protections under the Americans with Disabilities Act, an individual must have “a physical or mental impairment that substantially limits one or more major life activities” [42 U.S.C. § 12102(1)(A)].

Disabilities can manifest from birth or be acquired via an illness or accident. Many disabled people have a physical or cognitive impairment yet can generally be described as “healthy,” meaning that their condition and any functional limitations are relatively stable and predictable. However, illness is also a major cause of disability—think, for instance, of an individual whose mobility is impaired as a result of Parkinson’s disease. Individuals with acute illness or chronic disease may thus also identify as disabled (153) and qualify for accommodations under the Americans with Disabilities Act.

Disability can also be claimed as a cultural and/or political identity. Individuals with a wide range of medical diagnoses, phenotypes, and experiences have built a collective identity as people with disabilities. Many of the legal gains made by disabled people during the civil rights movement were the direct result of political mobilization born of collective identification. While some individuals view disability as a crucial part of their identity and a source of community, and therefore embrace the identity-first language of “disabled person,” others prefer the person-first language of “person with a disability” (5, 146). In what follows, we use both terms in an effort to reflect the variable preferences regarding language.

The relationship between genetic medicine and disability has been especially fraught due to the early entanglement of genetics and eugenics as well as the central focus of medical genetics on studying bodies that differ from a putative norm (76, 114, 137). Medical genetics developed in part by studying and characterizing intellectual disabilities and patterns of human “malformation” (73, 137), and the discipline continues to focus on characterizing and diagnosing conditions that are associated with physical differences and cognitive impairment (73). Disability advocates have criticized various genetic technologies and the practice of genetic counseling, calling attention to the dearth of disabled people’s voices in medicine, mistaken assumptions about quality of life for people with disabilities, poor communication about genetic diagnoses, a myopic focus on medical complications, and a problematic effort to eliminate not only genetic conditions but disabled people themselves (56, 138). These critiques have taken shape in and given rise to workshops (55, 56), advocacy organizations (56), communication guidelines (78, 131), and federal legislation (85).

This review surveys key conversations about the relationship of genetics and disability. We begin by situating contemporary genetics within a legacy of eugenics movements and then trace debates about the application of genetic technologies to prevent, predict, and treat disability, with particular attention to the implications of these critiques for genetic counseling and genetic medicine. We close by highlighting admirable efforts to shift how genetic counselors and medical educators address disability to suggest future directions for the field of genetic counseling.

## THE SCIENTIFIC LEGACY OF EUGENICS

The legacy of eugenics has shaped the contours of genetics, precision medicine, and genetic counseling, as these fields have had to reckon with the relationship between contemporary genetics and the eugenics movements of the nineteenth and twentieth centuries (30, 137). Though the particularities of eugenics movements differed across contexts, shared among these movements were the ideas that heredity was the source of much human difference and that the human species could be improved through selective breeding to eliminate undesirable traits. Eugenics was premised on claims about innate and heritable hierarchies between “races” of human beings that were shaped by racism and xenophobia (138). Proponents of eugenics advocated for state control over reproduction to bring about a superior human race, with efforts to eliminate putatively defective traits from the human gene pool and encourage the birth of healthy children.

The US eugenics movement of the nineteenth and twentieth centuries continues to be the subject of much historiography, the details of which are beyond the scope of this article (4, 30, 36, 64, 107, 138). Key for our purposes, however, is the fact that eugenics campaigns focused on the hereditary basis of human differences and sought to prevent the birth of people with impairments that are today termed disabilities. Commentators often differentiate between negative and positive eugenics, which took different approaches to pursuing a superior human gene pool. Negative eugenics used practices of sterilization, strict limitation on immigration, and laws preventing intermarriage of people of different “races” to prevent the birth of “defective” individuals. Conversely, positive eugenics promoted social programs to encourage “fitter” families to have more children (111).

Though the term disability was seldom deployed within the eugenics movement, many of the traits that eugenicists deemed undesirable and worthy of sterilization or institutionalization were conditions like blindness, deafness, intellectual disability, and mobility impairments, which are now commonly conceptualized as disabilities. As cogently detailed by Miller & Levine (80), eugenics campaigns, including laws aimed at restricting disabled people from being parents and the introduction of “prenatal testing recommendations predicated on the lesser worth of persons with disabilities” (p. 95), have contributed to historic trauma for people with disabilities.

Genetic science undergirded many eugenicist ideas about heredity. For instance, biologist Charles Davenport’s 1911 book *Heredity in Relation to Eugenics*—which Philip R. Reilly describes as “arguably the first textbook of clinical genetics” (111, p. 355)—theorized that many forms of intellectual disability were caused by “defective” genes and provided intellectual support to eugenicist policies of reproductive control. Efforts to eliminate people with these conditions were justified by the idea that such individuals were innately inferior and bearers of heritable defects that should not be passed along to future generations.

After World War II, eugenic ideologies and practices persisted but changed focus and scope. Eugenicists in the postwar period focused on individual choice and private decisions, as evidenced by a handful of heredity clinics and university-based human genetics departments that emerged in the 1940s (137). In contrast to the state-imposed control of reproduction that characterized twentieth-century eugenics, geneticists at these centers aimed to educate individuals about hereditary risks in order to help parents avoid having offspring at high risk of genetic conditions.

The practice of genetic counseling emerged within this context. Geneticist Sheldon Reed introduced the term genetic counseling in 1947 to describe “a kind of genetic social work without eugenic connotations” (110, p. 335). Reed studied the incidence of intellectual disability in families at the University of Michigan’s Hereditary Clinic and counseled prospective parents about their chance of having a disabled child. He believed in preventing disability by sharing information about genetic risk to impact the reproductive decisions of his clients. Genetic counselor Robert Resta describes how, in Reed’s view, “the goals of human genetics shifted from the financial and racial benefits for society to the medical and psychological benefits for families” (112, p. 376).

Accordingly, Reed considered genetic counseling to be “the process of helping families come to grips with the medical and psychological effects of genetic disease” (112, p. 376). He adopted progressive practices like clearly and compassionately communicating with parents to help them understand that they were not to blame for the conditions that affected them or their children. His approach is credited with empowering families to make their own decisions in a way that reflected societal trends of growing parental advocacy, as parents of disabled children were increasingly demanding better social support, medical attention, and options beyond institutionalization for their children (137). Although he played no direct role in establishing genetic counseling as a master’s-level profession, he is credited with developing a psychosocial approach and “patient-centered ethos” that informed the development of modern genetic counseling (112, p. 377).

Genetic counseling evolved into a distinct professional discipline several decades later with the founding of the first master’s program at Sarah Lawrence College in 1969, during a period that was characterized by key developments like the legal enshrinement of abortion rights, the advent of prenatal screening, the development of bioethics as an academic discipline, and the burgeoning disability rights movement (137). Genetic counselors in this era were typically women with advanced degrees who helped families adapt to genetic conditions and make decisions about reproduction and care (56, 137). Respect for patient autonomy was a guiding principle of genetic counseling, as it helped genetic counselors distinguish their role in reproductive decision-making from the state control of reproduction that characterized eugenics. The emphasis on autonomy and efforts to set genetic counseling apart from eugenics led to the ideal of nondirective communication, though this approach has increasingly been critiqued and replaced by a focus on the psychosocial components of genetic counseling (34, 126, 137).

## DEFINING DISABILITY: FROM THE INDIVIDUAL TO THE SOCIAL

The expansion of genetic technologies and the growth of the profession of genetic counseling coincided with the rise of the disability rights movement in the United States. Prenatal screening increasingly enabled prospective parents to test pregnancies for conditions that are associated with disability and to consider selective termination. At the same time, people with disabilities were mobilizing to overcome discrimination and bias and to have their lives viewed as worthwhile, as they demanded basic rights and called for broader shifts in societal attitudes toward disabled people (97). Disability activists and scholars called into question the dominant medical conception of disabled bodies as problems to be fixed, offering instead a social model of disability that encouraged individuals to see their challenges in life as resulting from barriers to social inclusion, from built spaces to social bias (100).

This shift in thinking about disability had—and continues to have—implications for medical practice, including medical genetics. Most disability-informed critiques of biomedicine distinguish between two frameworks for understanding the nature of disability: a medical model and a social model. Early critics of prevailing attitudes toward people with disabilities observed that medical providers—and the general public—see disability predominantly as a matter of individual deficit. Simon Brisenden (18) introduced the concept of a medical model of disability in 1986, characterizing the medical perspective as one that posits clinicians as the true authorities on disability and sees clinical diagnosis as foundational to understanding a disabled person. This emphasis on diagnosis leads to a “partial and inhibiting view of the disabled individual” (18, p. 173) and a tendency to focus on medical treatments rather than holistic well-being. In this view, an individual is seen as disabled because they lack certain physical or intellectual capacities necessary for pursuing a good life or participating fully in society.

As numerous scholars and advocates have noted, this medicalized notion of disability tends to stir feelings of pity—disabled people are seen as feeble, helpless, and unhappy—or perhaps admired for “heroically” overcoming the challenges posed by their disability. Narratives about people with disabilities in film, literature, and other media often instantiate this “tragic” imagination of disability, framing disabled people either as tragic victims or as heroes overcoming the supposed catastrophe of their bodily limitations (45, 99).

In contrast, what is often termed the social model of disability emphasizes that it is in large part society that disables individuals. Proponents of this perspective often rely on a distinction between impairment and disability. While many conditions or events can cause impairment, it is not always impairment itself that creates obstacles for well-being. Social biases, an inaccessible built environment, and inadequate social support are disabling in ways that transcend physical or mental traits or capacities. For instance, a deaf person is not disabled by their lack of hearing; rather, they are disabled by a hearing society that does not provide means of communication other than verbal speech. While the medical model focuses primarily on treating individual impairments or supposed deficits, a social perspective shifts the lens to examine the societal forces that make life more difficult for people with disabilities (100).

There are, of course, some impairments that do involve negative experiences that are closely tied to the impairment itself, such as conditions that cause physical pain or intense fatigue, and these experiences are not entirely explained by the social model of disability (48). Yet even in the case of such conditions, social factors worsen their inherently negative aspects. Someone with chronic pain might suffer from physical pain, but also from a lack of access to social events or steady employment due to that pain. Further, even these more “negative” impairments are generally not experienced as entirely or overwhelmingly negative (153). A social model of disability need not imply that medical treatment is never justified or desired—indeed, as Susan Wendell notes, “some very much want to have their bodies cured, not as a substitute for curing ableism, but in addition to it” (153, p. 18).

The term social model of disability was coined in a 1983 book by Mike Oliver (98)—a professor who later developed the first disability studies course—for a professional audience of social workers. The social model was intended to reorient attention to the role of oppression in the construction and experience of disability, not to serve as an all-encompassing framework for understanding the entirety of the experience of disability (48). Though the social model is occasionally presented as a totalizing theory (101), it can instead be understood as a lens that draws attention away from individual impairment and toward the sociopolitical aspects of disability (100).

Bringing a social lens to disability demands that we also attend to the ways in which some people with disabilities are multiply marginalized. Experiences of disability vary substantially based on social privilege or marginalization. Disabled people exist at multiple social locations, and it is crucial to think about disability from an intersectional perspective. Intersectionality, as articulated by legal scholar Kimberlé Crenshaw, is an attitude or lens that attends to the way that “various forms of inequality often operate together and exacerbate each other” (136). A social framework or model of disability must seriously attend to the multiple intersecting oppressions faced by disabled people of color, LGBTQI individuals, and other minoritized groups.

In the United States, racism has led to a society in which disabled people of color, particularly Black, Indigenous, and Latine individuals, experience structural and interpersonal racism and substantial socioeconomic disadvantage. A 2020 report released by the National Disability Institute showed that across all racial and ethnic groups, 26% of people with disabilities are living below the poverty line, compared with 11% of people without a disability, and that poverty rates are highest among Black and Indigenous people with disabilities (86). A social model of disability looks beyond medical treatment to structural injustices like socioeconomic disadvantage and systemic racism as central challenges for flourishing and equity for people with disabilities.

## PREVENTING DISABILITY: DISABILITY CRITIQUES OF PRENATAL SCREENING

Disability scholars and advocates have drawn upon the social model of disability to question the use of biomedical technologies that aim to prevent disability. If impairment or deviation from so-called normal functioning is not inherently bad and we abandon the idea that disability is inimical to well-being, what justification is there for endeavoring to eliminate it? This question has been central to debates concerning prenatal screening and genetic testing, which have been the focus of robust activism and scholarship.

In the 1970s and 1980s, alongside societal aspirations toward inclusion of people with disabilities, prenatal screening and diagnosis became an increasingly common part of prenatal care (56, 137). Cost-effectiveness was a pervasive justification of prenatal testing from the outset, as the resultant terminations had been shown to save the state and healthcare system money (137). While abortion had been available in some states prior to the *Roe v. Wade* decision in 1973, this historic decision marked the legalization of abortion across all states and led to much wider (though by no means universal) availability of safe abortions (49). This meant that a prenatal diagnosis of a genetic anomaly could, and frequently did, lead to termination (137).

From the perspective of many disability rights advocates, selective termination based on genetic diagnosis is, in essence, a means of disability prevention—a means of solving the “problem” of disability (7, 152). Put bluntly, “it prevents disability by simply killing the fetus” (106, p. S3). However, if disability does not inherently impair quality of life, and if life with a disability can be just as worthwhile as a life without disability, then the moral justification for terminating fetuses with a disability weakens. Adrienne Asch, a social psychologist and bioethicist who became blind as an infant, was a leader of what came to be known as the disability rights critique of prenatal screening. In 1999, Asch and Erik Parens published an influential article in the *Hastings Center Report* that characterized the disability critique as comprising two central lines of argument (106). One argument focused on the immorality of selective termination, while another emphasized that selective termination on the basis of genetic diagnosis was often based on misinformation.

Some critics of selective termination on moral grounds suggest that the practice encourages a problematic relationship between parent and child, reflecting a desire for perfection that is detrimental to the unconditional acceptance that should characterize parental love (106). Others make the expressivist objection, which claims that selective termination expresses negative views of people who carry a disabling trait and, in so doing, devalues the lives of disabled people (106). Recent empirical research has found that individuals and families living with genetic conditions articulate concerns that echo the expressivist objection—for instance, worrying that termination based on genetic diagnosis could be expressive of a statement about the value or worthwhileness of life with a genetic condition, or could imply a recommendation about the right course of action for others (13). A 2018 study by Felicity Boardman and Rachel Hale found that individuals who more strongly identified with their disability held more negative views of selective termination (16). Yet other studies have found more positive attitudes and general support for prenatal diagnosis among people with disabilities (11, 28).

Today, the landscape of reproductive health in the United States has shifted in the wake of the Supreme Court decision in *Dobbs v. Jackson Women’s Health Organization* to reject a constitutional right to abortion. Access to abortion is becoming increasingly restricted, and the freedom to choose to terminate a pregnancy has been reduced. Though some anti-abortion activists appeal to disability rights to advocate for abortion bans of selective termination based on fetal diagnosis, many disabled people affirm the right to abortion, oppose efforts to eliminate reproductive freedom, and seek to ensure that all people—including people with disabilities—have access to reproductive healthcare and unbiased information (2). Disability rights and pro-choice advocacy are not incompatible and have, in fact, frequently been undertaken simultaneously.

Asch, a vocal advocate for both disability rights and women’s rights to abortion, was a strong proponent of the idea that selective termination is often the result of misinformation. She supported the freedom to choose whether to move forward with a pregnancy but argued that the decision to terminate should be fully informed and not based in bias against disabled people. In her view, medical providers overly emphasize the negative aspects of disability when communicating about reproductive choices and a potential child’s future life, suggesting that the lives of people with disabilities are of lower quality than other lives (56). As she puts it, when “life with disability is described at all, it is usually a description filled with gloom and tragedy” (56, p. 164). Such descriptions misrepresent life with a disability, and reproductive decisions based on misinformation are not truly informed.

These formative critiques of prenatal screening have evolved with technological developments in both prenatal and reproductive screening and diagnostic testing. With serial advancements in maternal serum screening, cell-free fetal DNA testing, and expanded carrier screening, prenatal and reproductive screening and diagnosis have become more common and now include testing for a growing number of genetic conditions that are associated with a wide range of conditions (69, 81). Importantly, these trends raise concerns about the impact of the routinization and commercialization of prenatal screening and reproductive genetic testing on informed decision-making (81). Whereas historically, providers’ attitudes were a primary focus of disability critiques of prenatal testing, widespread commercialization of testing raises concerns about potential biases in laboratory education materials about tests and conditions tested (81) as well as potential conflicts of interest for genetic counselors employed by genetic testing laboratories (140). Nonetheless, physicians and genetic counselors continue to play a critical role in communicating about disability, and their organizations influence professional norms and recommendations with respect to prenatal and reproductive testing.

## COMMUNICATING ABOUT DISABILITY: ASSUMPTIONS AND BIAS IN REPRODUCTIVE COUNSELING

Critiques that medical providers and prospective parents are ill-informed about what it is actually like to live with a disability have important implications for clinicians, especially given that such concerns are substantiated by empirical studies. In a 2021 survey of 714 practicing US physicians, 82.4% reported that people with significant disability have worse quality of life than people without disability (58). Yet this assumption is not borne out by the self-reports of people with disabilities. A substantial body of research shows that disabled people generally report having good or excellent quality of life, on par with or only slightly lower than self-reports of nondisabled people (12, 149). This finding has been deemed so surprising as to garner its own term, the disability paradox, to name the ostensibly perplexing fact that so many people with disabilities report high levels of life satisfaction while observers assume that they are dissatisfied with their lives (3). Of course, this is only a paradox within a framework that automatically associates disability with poor quality of life (25).

Within the prenatal and reproductive testing landscape, the perceptions of medical providers of the severity of different genetic conditions have played a role in the design of expanded carrier screening panels. As several scholars have noted, the American College of Medical Genetics and Genomics’ clinical practice resource on expanded carrier screening defined the severity of genetic conditions based on a single study performed by a commercial laboratory that relied on the perceptions of genetic counselors, geneticists, and physicians, without consultation with individuals with lived experience of the conditions in question (69, 82, 139). Importantly, the National Society of Genetic Counselors (NSGC) practice guideline on expanded carrier screening articulated the difficulty in defining condition severity and called for the amplification of perspectives from the disability community during discussions of expanded carrier screening panel composition and policy decisions (120).

These considerations are further elucidated by a growing body of research investigating the perspectives of people with genetic conditions and their family members on condition severity. In 2021, Felicity Boardman and Corinna Clark performed a qualitative study of the concept of seriousness from the perspective of individuals with a range of conditions that are commonly included in carrier screening and are often considered clinically serious, such as fragile X syndrome, spinal muscular atrophy, and cystic fibrosis (15). They found that most participants reported good health and well-being and the capacity for good quality of life. Individuals with later-onset conditions were more likely to view their condition as an illness and to hold more negative views of their quality of life than those with early-onset conditions, who were more likely to see their condition as part of their identity. Across groups, the existence of social support was a significant mediator of quality of life, with participants who felt unsupported by society reporting lower quality of life. Empirical research has also shown that families that include disabled children fare on average no better or worse than families in general (42, 106). Yet much communication about genetic diagnoses in both prenatal and postnatal contexts presupposes lowered quality of life for people with genetic conditions and their families.

The way a diagnosis is communicated—from the phrases a provider uses to the additional information provided (or not provided)—can have a significant impact on how parents feel about the diagnosis. Provider–parent conversations about genetic diagnoses are emotionally laden, often prompting feelings of shock and grief (51, 54). When parents receive a prenatal diagnosis, they must rapidly assimilate complex medical information and contemplate raising a child with a disability, and then make life-altering decisions about whether to continue the pregnancy (54). Communicating about genetic conditions always involves making a choice about what story to tell, and the choice of narrative framing may influence attitudes and behaviors (74).

Down syndrome (DS) has received particular attention in studies on provider communication. Most parents of children with DS state that their child has had a positive impact on their lives, and the majority of people with DS report a high quality of life (6, 127). Yet provider communication about the diagnosis tends to emphasize the negative aspects of DS, exacerbating parental distress. In a study of parent experiences of prenatal DS diagnoses made between 2016 and 2021, parents of children with DS recalled that obstetric gynecologists and maternal fetal medicine specialists were more likely to discuss medical issues and reproductive options than support, services, and psychosocial outcomes. Respondents in this study reported that 61% of these providers had framed it as bad news (e.g., saying, “I’m sorry”) and that physicians who framed it as bad news were less likely to provide information on support, services, broader life outcomes, and condition-specific resources (79). Similarly, in a 2024 study, a substantial number of mothers who were given postnatal diagnoses of DS reported that providers emphasized the negative aspects of DS and omitted positive aspects, with only 24% of parents reporting receipt of adequate explanatory materials (6).

While providers and parents alike note the importance of providing information about genetic causes, medical issues and developmental delays, and therapies and advocacy organizations, parents also indicate that they especially value information that demonstrates the abilities, potential, and positive impact of children with DS. Parents want—and are often not given—information about what life is like with a child with DS (122). This is problematic given that informed consent cannot be secured without parents receiving fulsome information about what it is like to live with or care for someone with DS (106).

Genetic counseling sessions offer an opportunity to educate parents about testing options and specific genetic conditions (106). Best practices for offering prenatal screening consistently recommend genetic counseling, and research shows that patients report better affective and knowledge outcomes when genetic counseling is provided as a part of prenatal screening (81). Genetic counselors are specifically trained to provide balanced and up-to-date information about genetic conditions and to use sensitive, neutral language (131). They are also increasingly attentive to the importance of word choice (124) and patient preferences when discussing disability (71). Despite these important strengths, research led by genetic counselors and others demonstrates that parents do not consistently receive robust information about lived experience with particular genetic conditions when meeting with genetic counselors (41, 116). Research further suggests that genetic counselors, like other medical providers, harbor implicit biases against people with disabilities (52) and that prenatal genetic counseling often frames disability with an emphasis on negative aspects of disability and medical impairments, with limited attention to positive aspects of disability or social considerations (116).

DS has again received the most attention in empirical studies of this phenomenon. Ijaz et al. (60) studied parent recall of information provided in a genetic counseling session to assess the degree to which genetic counselors adhered to communication guidelines for delivering a DS diagnosis. While NSGC developed a practice resource for communicating a prenatal or postnatal diagnosis of DS in 2011 (131), one study found that parental reports indicated that genetic counselors covered slightly less than half of the topics recommended in that resource. Notably, respondents had more positive diagnostic experiences of genetic counseling sessions that they recalled as having higher perceived practice resource adherence (60).

NSGC’s development of guidelines on communicating about DS and the existence of robust patient information resources reflect advocacy by disability activists as well as clinicians to ensure balanced communication about disability. That greater adherence to NSGC practice guidelines on communicating a DS diagnosis and the provision of information about broader life outcomes were associated with more positive diagnostic experiences speaks to the value of both communication guidelines and robust informational resources. The Lettercase National Center for Prenatal and Postnatal Resources offers up-to-date, balanced information about DS and other genetic conditions, making expertly designed materials readily accessible (148). While the Prenatally and Postnatally Diagnosed Conditions Awareness Act was passed in 2008 to promote the provision of accurate information about DS at diagnosis, lack of funding and enforcement has limited its impact (85), leading many states to pass similar laws that vary significantly (72, 81). Despite this, clinicians are inconsistent in their adherence to communication guidelines and their provisioning of robust information about genetic conditions.

Additional developments in prenatal and reproductive genetic technologies—such as preimplantation genetic diagnosis, gamete donor screening, and polygenic embryo screening—continue to spark debate about disability, quality of life, patient–provider communication, unregulated commercialization, and responsible uses of genetic technologies. Cases in which hypothetical parents use preimplantation genetic diagnosis to select for genetic conditions associated with disability have gained particular attention and generally focus on selecting for conditions like achondroplasia or deafness (50, 141). Robust philosophical arguments highlight the different views that affected individuals and clinicians may have about what constitutes a “good life” and emphasize that reflective decisions about using preimplantation genetic diagnosis generally fall within the boundaries of parental authority, even though professional guidelines state that providers can ethically permit or decline such requests (31).

## DIAGNOSING DISABILITY: PROGNOSTICATION AND POSSIBLE FUTURES

That most activism and research on disability and genomics have focused on prenatal and reproductive contexts is limiting given that genetic testing and counseling are increasingly implemented across the life span. From neonatal screening and pediatric diagnostics to specialized adult centers devoted to neurogenetics, cardiac genetics, and cancer genetics, genetic testing and counseling impact individuals and families in myriad contexts and life stages.

Research on the utility of genetic testing in neonatal and pediatric contexts has demonstrated how diagnoses in a variety of patient populations, including those with neurodevelopmental and other conditions associated with disability, can impact patients and families. Genetic test results can inform precision therapies, tailored referrals, support and services in community care contexts, and anticipatory guidance for patients and families (20, 23, 133). However, clinicians can also interpret and communicate genetic diagnoses in ways that foreclose aggressive therapy (22, 62, 123), by limiting surgical intervention or respiratory support (56, 65) and redirecting care toward palliation for infants with different types of genetic diagnoses (26). Stakeholders and researchers have therefore raised concerns that clinicians may inappropriately use genetic diagnoses to counsel against interventions and thus create a self-fulfilling prophecy (65, 123).

One study employing a series of vignette-based experiments found that neonatologists were more likely to recommend palliative rather than intensive care for infants with genetic findings associated with neurodevelopmental disability, despite the variability in these conditions (23). Furthermore, genetic testing in neonatal intensive care rarely leads to specific treatments and instead provides prognostic information—that is, information about what to expect for a child’s future and the natural history of the diagnosed condition (23). Since the prognostic implications of many genetic diagnoses are variable, uncertain, and evolving, this raises important questions about how providers and parents interpret and communicate about the prognostic uncertainty associated with genetic findings that may be associated with disability (23).

Amid this shift toward genotype-first diagnostics, the sociologist Daniel Navon observes that genetic counselors often become activists for novel genetic conditions that have uncertain implications and that are poorly characterized (95). In this role, genetic counselors help “to create new categories that will shape expectations and treatment regimens for both existing patients and those who are yet to be diagnosed” (95, p. 770). This type of activism holds significant promise to help patients and families by connecting them and better defining conditions over time but also raises the question of whether genetics is always “the best rubric for understanding a person’s developmental disability or difference” (95, p. 774). Therefore, as microarray and whole-exome sequencing are increasingly ordered irrespective of clinical presentation, it is especially important for genetic counselors to emphasize “the indeterminate developmental potential of every child” in advocating for genetic conditions associated with disability (95, p. 770).

While there is research investigating how diagnostic and prognostic information about genetic diagnoses is delivered to parents in prenatal and pediatric settings, much less is known about how children diagnosed with disabling or life-limiting genetic conditions learn about their diagnoses and prognoses as they grow up and become adults. Genetics has historically prioritized prenatal and pediatric diagnostics, so parental perspectives have garnered greater attention than the perspectives and experiences of adults growing up with these conditions. Insomuch as biased and outdated portrayals of genetic conditions are common, greater attention to the lived experience of prognosis may help clinicians and researchers better understand the impact of predicted and progressive disability and cultivate disability cultural competence (46, 84).

While genetic diagnoses can unnecessarily limit expectations, they can also enable profoundly important and influential types of identity and community, in addition to tailored medical care. Social scientists have used the term biosociality to describe the ways in which biomedical knowledge shapes social identity and relationships (47, 109). The construction of social identities, relationships, and communities based around biomedical knowledge and diagnoses is especially apparent in genetics, where the discovery of a genetic etiology can lead to new clinical entities that inspire support groups and the establishment of condition-specific foundations (95, 96). Convening around genetic diagnoses enables people with genetic conditions and their family members to build collective knowledge, which can augment or offset challenges associated with exclusively medical models of disability (56, 143). Thus, while some disabled people push back against genetics, others build identity and community from genetic diagnoses.

## TREATING DISABILITY: DISABILITY IDENTITY, CULTURE, AND THE PURSUIT OF “CURES”

As the capacity to intervene in genetic conditions across the life span continues to develop, genetic counselors, geneticists, and other physicians face an increasing need to be aware of novel therapies (150, 151). Whether genetic counselors are helping an individual or family make sense of a genetic diagnosis that is associated with disability or counseling prospective parents about a genetic condition, awareness of the shifting treatment landscape is critical because novel treatments may alter prognostic implications (158).

While some people with genetic conditions that are associated with disability want novel treatments like gene therapy, genetic counselors and other clinicians should also be aware of cautionary and critical perspectives on different treatments within disability communities. Gene therapies are generally touted as treatments for genetic diseases, not disabilities. However, many genetic diseases cause impairments that fall within the Americans with Disabilities Act’s definition of disability, such as mobility impairments and medical and intellectual disabilities. In addition to meeting the legal criteria for disability benefits, individuals living with genetic conditions may at some point in the course of their illness begin to identify as disabled or as a person with a disability. Thus, emerging gene therapies that enable intervention at the level of the genome raise important questions about when it is ethically and socially advisable to seek to “cure” conditions associated with disability (142) and whether resources are better allocated toward individual treatments or the removal of structural barriers to disability access (82).

Scholars and advocates have argued that efforts to cure or eliminate certain conditions, such as heritable deafness, achondroplasia, and autism, reflect ableist ideas about the inferiority of life with a difference understood as a disability. Philosopher Elizabeth Barnes (10) argues that disabilities can constitute a “mere difference” rather than a “bad difference,” asserting that disability does not intrinsically make people worse off or prevent well-being. The desire to find a “cure” for disabilities is in many instances rooted in the idea of disability as a bad difference rather than a mere difference and a valuable form of human diversity. Many people resist this bad-difference framing and embrace disability as a meaningful and positive component of their experience of life and selfhood (29).

Deafness is often used as an exemplar in this debate. Since the introduction of cochlear implant technologies in the 1980s and 1990s, some deaf people have rejected the search for a cure for deafness (68). Those who participate in Deaf culture through the use of sign language and visual communication often consider deaf individuals to be part of a linguistic minority and affirm deafness as a valuable and positive identity (129). Some have argued that cochlear implants, which seek to make deaf children into hearing children, are a harmful attack on Deaf culture, even a form of genocide or colonialism (104, 135, 157). Like cochlear implants, the use of genetic technologies to eliminate deafness can be seen as a medical overreach that fails to acknowledge the reality and value of Deaf culture and Deaf identity (103).

A similar debate has unfolded regarding achondroplasia, a genetic condition caused by a mutation in the *FGFR3* gene. As with deafness, some people with achondroplasia value their condition as a part of their identity, embrace “dwarf pride,” and may oppose the search for medical cures (such as gene therapy) that focus on normalizing their height (134, 155). Little People of America, an organization that provides support and information to people of short stature and their families, opposes treatments focused solely on growth velocity but are in favor of research focused on treatment outcomes, like reducing spinal stenosis and the need for corrective surgeries (75).

Even adults and parents of children living with genetic conditions less readily associated with a minoritized culture do not always embrace the search for cures. One study of adults and parents of children diagnosed with spinal muscular atrophy found that some participants expressed ambivalence about a novel targeted therapy (nusinersen) and viewed it through a disability lens (102). Some adults with spinal muscular atrophy regard their condition as integral to their identity and have expressed that taking nusinersen conflicts with disability pride (77) and that the money spent on the novel therapies would be better directed to services to enable independent living (102). Perspectives on treatment vary within and across conditions, with research indicating that people who experience forms of disability that involve pain, deterioration, and a shortened life span are more likely to embrace a cure than those living with impairments that are relatively static after birth (14, 130). Notably, the common rhetoric of “cure” can imply a complete elimination of a condition, but in reality, the results of gene therapies are variable and may include severe complications (8).

Given that most genetic conditions are variable and that experiences of impairment and disability are diverse, attitudes toward treatments, appropriate endpoints for clinical trials, and cures may vary (82, 93, 115, 118). It is therefore crucial for research to integrate community values and priorities to assess whether treatments are desired and to identify what patients and families perceive to be meaningful benefits and tolerable side effects (61, 115). The development of patient-centered disease concept models (154), treatment preference research (61, 67), and empirical and conceptual work aimed at integrating community values and priorities into studies of human gene editing (115) may all provide useful frameworks for responsibly incorporating the perspectives of people with disabilities and their family members into research aimed at developing novel therapies for genetic conditions. Additional relevant perspectives can be found in a growing body of literature on disability, genetics, and precision medicine that calls for anti-ableism (34, 82) and offers models for involving stakeholders in all phases of research (82, 93, 118). Ultimately, stakeholder research must therefore also grapple with the normative stakes of their findings, considering not only what people with genetic conditions think but also how researchers and clinicians should proceed given these heterogeneous opinions.

## DISABILITY EDUCATION, ADVOCACY, AND RESEARCH IN GENETIC COUNSELING

Given their training and scope of practice, genetic counselors may be especially well-positioned to incorporate the perspectives of disabled people into genetic medicine. Genetic counselors are guided by NSGC’s relationally based professional code of ethics (89), which stipulates that genetic counselors have ethical obligations to society more broadly, including engagement in activities that promote access to healthcare services and prevent genetic discrimination. Given this explicit commitment to addressing societal issues related to genetics and their frequent contact with people with disabilities and their families, it is imperative that genetic counselors attend to disability accessibility and accommodation, stigma and unconscious bias, and language and communication (71, 119).

Genetic counselors have periodically addressed the tensions and connections between the disability and genetic counseling communities (76, 113) and advocated for shifts in language choice, education, research, and public policy (76). Genetic counselors have also facilitated research related to the provision of genetic counseling and testing to patients with different disabilities (9, 21, 38, 43, 44, 105) and worked closely with communities affected by genetic conditions that are associated with disability (56, 95). For example, in the 1980s, the genetic counselor Beth Fine, Dr. John Carey, and parents of children with trisomy 18 worked together via the Support Organization for Trisomy to develop educational materials that offered humanizing images of individuals with trisomies and information on treatment options in an era when most clinicians dismissed the condition as lethal (56). Nonetheless, there are few guidelines or standards for consistent education about disability in genetic counseling.

For genetic counselors to effectively engage with disabled individuals and work against ableism, they must undergo robust and consistent disability education and training with clear leadership from professional organizations and accrediting boards. Identifying and correcting biases regarding people with disabilities should be an important part of the education of genetic counselors and other clinicians. This is especially important given that researchers have already begun to demonstrate that anti-bias training is effective and can help clinicians offer appropriate genetic counseling recommendations in prenatal contexts (63).

Genetic counseling educators have long been pioneers of disability-informed medical education. Brandeis University’s genetic counseling program was an early leader in disability education (56, 137). Founded in 1992 by Judith Tsipis, a biologist whose son Andreas had Canavan disease, the program had a distinguished advisory board that included disability scholars and self-advocates like Kennith Irving Zola and Marcia Saxton, who made critical contributions to the education and consciousness-raising of genetic counseling students and practicing genetic counselors (56). At Brandeis, disability awareness and advocacy were central features of genetic counseling. Trainees took an annual seminar centered on learning from disabled adults with genetic conditions and participated in a program that facilitated close engagement and social activities with disabled children and their families. Other programs, like those at Northwestern University; the University of California, Berkeley; and Johns Hopkins University/NIH also made early efforts to integrate community engagement and disability perspectives into the genetic counseling curriculum. Early innovations to disability education in genetic counseling were often inspired by genetic counseling educators’ immersion in condition-specific communities or contact with Adrienne Asch and other disability scholars and advocates (56).

More recently, genetic counselor Devin Schuman’s work has focused on training clinicians to critically examine common conceptions and representations of disability (125). Schuman asks genetic counselors and other clinicians to question culturally ingrained images of disabled people, such as notions of people with disabilities as tragic figures in need of pity or heroic warriors to be admired, and offers concrete steps that genetic counselors and other clinicians can take to “pave the path to inclusivity,” focusing on a variety of “quick gut-checks for ableist biases” and language to avoid and to consider using (124). She also encourages clinicians to be critical consumers of disability media and to wisely choose resources and language when communicating with individuals and families about actual or potential disability (125).

Affirming representations of disability are now common, which may help clinicians and the public to overcome biases. Instagram and TikTok personalities regularly challenge assumptions about disabled bodies as different or foreign by sharing their everyday lives, showing makeup tutorials, lingerie, and tracheostomy tubes as all part of a happy life. Sins Invalid, a performance project committed to disability justice, encourages art that challenges paradigms of bodily “normalcy” and makes an “an unshamed claim to beauty in the face of invisibility” (132). Even the *New York Times* developed a regular column, “About Us,” dedicated to essays written by people with disabilities.

Genetic counselors with disabilities are also increasingly sharing their own experiences of disability, calling attention to disability as an important and neglected form of diversity within the genetic counseling profession (57, 124). While NSGC has been making significant efforts to increase the racial and ethnic diversity of the genetic counseling profession, less attention has been paid to ableism within the profession. Rather than limit disability discourse and advocacy to questions of patient care, genetic counselors are voicing a need to support and include genetic counseling colleagues with disabilities. Through articles in NSGC’s *Perspectives* newsletter (https://perspectives.nsgc.org) and blog posts at *The DNA Exchange* (https://thednaexchange.com), genetic counselors with disabilities are detailing experiences of exclusion within the profession, sharing challenges with gaining accommodations, and detailing experiences of ableism within genetic counseling and medicine more generally (24, 53, 57, 83, 117). In 2020, disabled genetic counselors convened and formed DisabilityGC, an informal working group that meets quarterly and maintains an email distribution list (24). These community-building efforts have led to research on the experience of disabled genetic counselors (39), multiple NSGC-sponsored talks, the development of a mentorship program for disabled genetic counseling students (24), and accommodation guidelines for genetic counseling students and programs (35).

While some genetic counseling programs have heavily incorporated disability perspectives into their curricula, educational standards for genetic counseling programs are more limited. In 2013, the Accreditation Council for Genetic Counseling (ACGC) incorporated awareness of disability into its diversity and equity instructional content standards for genetic counseling training programs (1). In a 2019 report titled *Genetic Testing and the Rush to Perfection*, the National Council on Disability urged ACGC to implement substantive disability education focused on social and cultural aspects of disability (85), but the standards remain limited (121). Consequently, curricular coverage of disability in genetic counseling programs varies widely. Programs report anywhere from 10 to 600 hours of relevant content in formats ranging from attendance at disability-related support groups to workshops on topics like appropriate language and the history of disability (121).

Nascent research on disability education in genetic counseling training has begun to assess educational priorities for genetic counselors from the perspectives of the disability community, including suggested curricular content (37), potentially effective pedagogical approaches for disability education, and educational needs of genetic counselors with disabilities (32, 37, 39, 156). This work suggests that genetic counselors and individuals with disabilities believe that equipping genetic counselors to provide patients with medical, social, and lifestyle aspects of disability is most important and that exposure to the everyday lives of disabled people is the most meaningful method of education (37). Lettercase, noted above for its provision of balanced information on genetic conditions, is now housed within the genetic counselor–led Genetic Support Foundation and offers scaffolded training modules for teaching healthcare providers how to communicate a DS diagnosis (148).

Many genetic counselors and genetic counseling educators already engage in clinically based disability advocacy, education, and disability-informed research, yet NSGC has historically been less active with respect to disability. In 2011, Madeo et al. (76) aptly detailed how NSGC has been politically active with respect to abortion rights and causes without similar advocacy or public statements about disability rights or disability causes. Since then, NSGC has developed guidelines (131), position statements (90), a conversation series highlighting issues related to disability (92), and materials to promote access to both genetic services and balanced information about genetic conditions (33).

More recently, NSGC has expanded its focus on and efforts around diversity, equity, inclusivity, and justice, and its flagship publication, the *Journal of Genetic Counseling*, has articulated a commitment to diversity in scholarship. This move reflects increased recognition and efforts to ameliorate both racial and ethnic disparities in genetic medicine (70) and the homogeneous demographics of the genetic counseling profession (40). Additionally, in September of 2024, NSGC adopted an updated position statement on disability that strives for accessibility, denounces ableism, endorses the right to self-determination for all individuals, and reinforces the importance of providing balanced “medical and social information about disability” (91). These are important developments, particularly in light of NSGC’s limited history of advocacy for disability (56, 76).

Given the National Council on Disability’s concerns about broadened use of prenatal screening tests (85), the development of polygenic embryo screening (94), the rise of genetic and protein-based therapies, and the NIH’s expressed commitment to addressing ableism (19), it is an especially critical time for the field of genetic counseling to operationalize its articulated commitment to supporting individuals with disabilities. Both NSGC and ACGC have important choices to make about political advocacy, public alignment with disability causes, and the potential expansion of educational guidelines and profession-based competencies related to disability. These organizations have an opportunity to build on the commitments and expertise that many genetic counselors have demonstrated in their disability-informed work over the last 50 years.

## CONCLUSION

Genetic counseling has a unique relationship with disability, given its role in prenatal screening and its focus on supporting patients and families living with genetic diagnoses that are associated with disabilities. Genetic counselors play a crucial role in interpreting genetic information, discussing prognoses, and helping individuals and families navigate healthcare decisions. They also help individuals and families adapt to the medical, psychological, and familial impacts of genetic conditions.

It is especially important, then, that genetic counselors and other clinicians are trained in communication and care that does not implicitly devalue disabled lives by presupposing common but inaccurate ideas about disability as unfortunate, tragic, or inherently linked to suffering and low quality of life. While genetics professionals have done important work to promote the provision of balanced information about genetic conditions and often lead efforts to support patients and families affected by conditions that are associated with disability, there is more to be done to ensure that genetic counselors and other medical providers consistently incorporate a disability-affirming lens in their communication and care of people with disabilities.

Acknowledging and challenging personal assumptions about the lives of people with disabilities is a necessary but insufficient step toward disability justice in genetic medicine (82). Researchers and disability advocates have highlighted both individual biases and structural contributors to disability discrimination in medicine, such as a minimally regulated genetic testing industry and bureaucratic barriers to accessing social security disability income. People with disabilities who also experience other forms of social marginalization experience particularly acute challenges for well-being and health. Genetic counseling and medical education must incorporate intersectional frameworks that foreground how ableism interacts with and is worsened by other forms of oppression, such as racism, classism, and gender discrimination.

As genetic counselors are generally skilled in communication and empathy and knowledgeable about genetic testing, they are well-poised to develop disability education standards and practice-based competencies. By building on the disability-informed work of genetic counselors and recent calls for anti-ableism and disability inclusivity in medicine, the field of genetic counseling can establish a broad, nuanced, and public commitment to advocacy for individuals and families living with disability. While discussions of genetics and disability have tended to focus on the reproductive context, there is a need for more attention to and resources for people living with disabilities who may themselves be pursuing parenthood, supportive communities, social services, and tailored medical care. By engaging with and advocating alongside the communities they serve, genetic counselors and other medical providers may come to understand how disability can be consistent with—and even a source of—joy and flourishing (45).

## Abbreviations

Identity-first language: language that expresses a view of a trait, condition, or disability as an inherent part of an individual’s identity, such as “Autistic person” or “Deaf person”
Person-first language: language that puts a person before a diagnosis, such as “person with a disability” or “person with hemophilia”; healthcare providers commonly use person-first language to emphasize that a person is not defined by their medical diagnosis
Social model of disability: a model for understanding disability that emphasizes how people are disabled by barriers in society and focuses on identifying and eliminating barriers to social participation
Medical model of disability: a framework that focuses on individual medical problems or physiological differences as the source of disability and emphasizes the need for medical intervention and treatment
Ableism: implicit or explicit discrimination against people with disabilities, which may be reflected in individual, societal, and institutional attitudes, norms, and policies
Disability paradox: the phenomenon whereby disabled people report a high quality of life while others assume they have a reduced quality of life
Disability cultural competence: a term used by disability theorist and bioethicist Rosemarie Garland-Thomson for “the promotion and development of bioethical, cultural, technological, and legal supports for people living with disabilities as they are” (46, p. 334)
Anti-ableism: actively opposing and challenging ableism to dismantle a system of discrimination and prejudice against people with disabilities
